# Remote Mock OSCE (ReMO): The “new normal”?

**DOI:** 10.1192/bjo.2021.368

**Published:** 2021-06-18

**Authors:** Rachel Brown, Elsa Brew-Girard, Stephen De Souza

**Affiliations:** Taunton & Somerset NHS Foundation Trust, University of Bristol

## Abstract

**Aims:**

In March 2020, COVID-19 and its associated restrictions forced a halt to in-person teaching and assessment. To try and mitigate this disruption, the psychiatry undergraduate teaching faculty developed a knowledge based remote curriculum. However, it became clear that our students sorely missed clinical and consultation experience. Prior to the pandemic we had delivered a mock Objective Structured Clinical Examination (OSCE) to those undertaking their psychiatry block. In Somerset Academy, we wanted to deliver a distanced alternative: the remote mock OSCE (ReMO). We hoped to demonstrate this would be a feasible and valuable learning experience.

**Method:**

In keeping with other OSCEs, ReMO had active stations (4) and a rest station. Four simultaneous Skype meetings were set up as clinical stations, each with an examiner and actor. To test the technology, students and facilitators were emailed links to each meeting in advance, and invited to sign in. Students were given individualised timings to rotate between stations. Stations involved history taking, risk assessment, and management discussions of common psychiatric presentations.

The students then rotated again, receiving personalised feedback about their performance, enabling immediate reflection and consideration of areas for development. This was followed up with written feedback, using examiner completed mark schemes.

**Result:**

After ReMO we invited feedback from medical students and facilitators. 7 out of the 8 medical students that participated completed a post-ReMO survey. 100% of students found ReMO “useful”, with 71% (5/7) rating it an “extremely valuable” experience and 29% (2/7) rating it “fairly valuable”. Students felt it was well organised, realistic, and increased their confidence in remote consultations and OSCE practice. 6 out of 8 facilitators completed feedback on ReMO. 100% felt that ReMO was reproducible and 83% (5/6) rated it as “fairly realistic” when compared to the face-to-face standard.

**Conclusion:**

Firstly, ReMO was feasible. However, it was logistically difficult, requiring extensive organisation to ensure this relatively small group were in the right place at the right time. In future, we would consider alternative platforms such as Zoom, or specific consultation software, such as Attend Anywhere, to reduce the logistics burden and utilise features such as ‘breakout rooms’. We would recommend an allocated co-ordinator to troubleshoot any problems in real time via a group messaging service.

In conclusion, ReMO is achievable and a valuable student learning experience. Since the pilot it has become an integral part of our curriculum. We recommend that all undergraduate Psychiatry faculties consider adding it to their programme.

